# Supplementary material for: An Oligopeptide-Protected Ultrasmall Gold Nanocluster with Peroxidase-Mimicking and Cellular-Imaging Capacities
Source: Molecules. 2022 Dec 21;28(1):70. doi: 10.3390/molecules28010070 (PMC9822283; doi:10.3390/molecules28010070)
Supplement: Supplementary file 1 [file molecules-28-00070-s001.zip › molecules-2063630-supplementary.pdf]

An Oligopeptide-Protected Ultrasmall Gold Nanocluster with Peroxidase-Mimicking and Cellular-Imaging Capacities

Supplementary Information

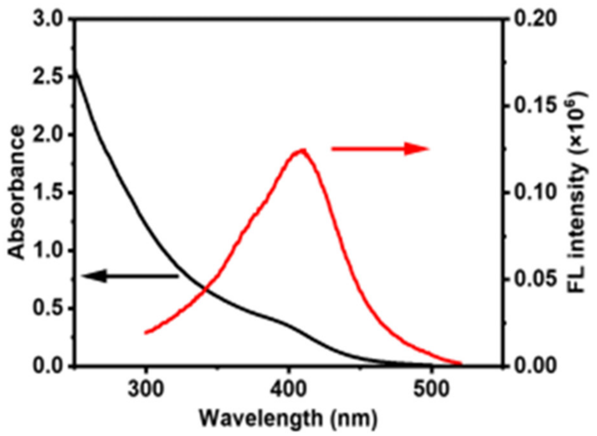

Figure S1. UV-vis absorption spectrum (black line) and the luminescence excitation (red line) of PGN.

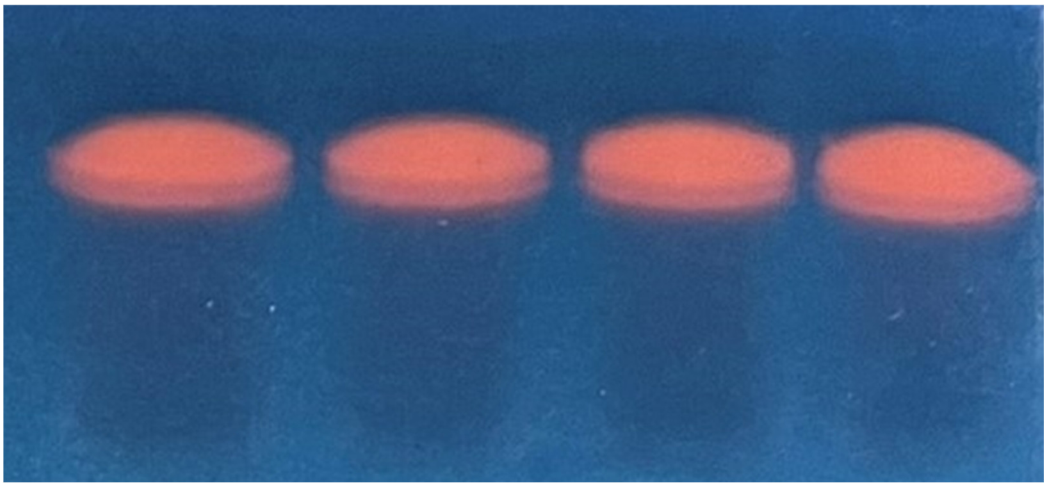

Figure S2. Digital photo of the PAGE bands upon UV light excitation.

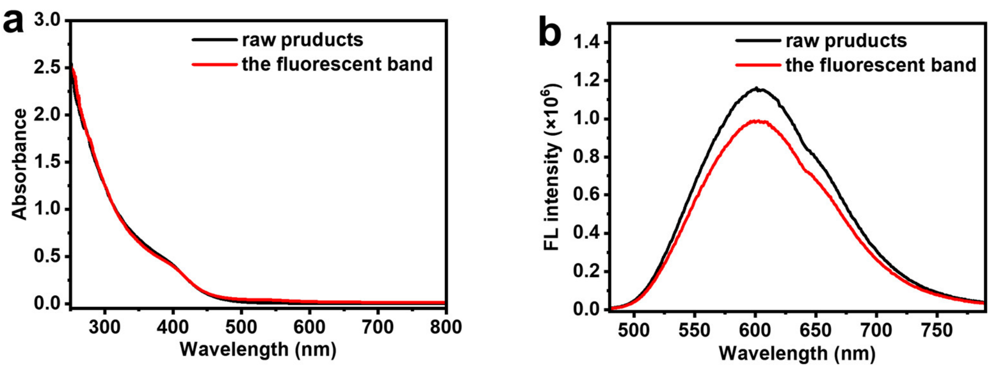

Figure S3. Comparison of (a) UV-Vis and (b) fluorescence intensity of the crude products and the fluorescent band.

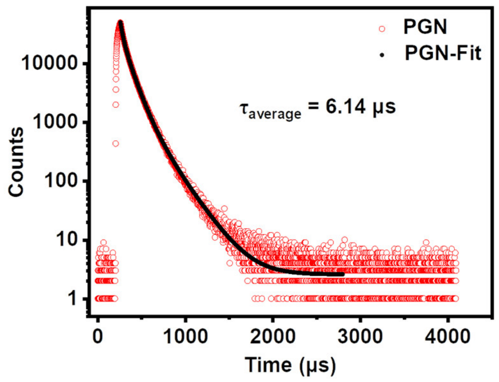

Figure S4. Fluorescence lifetime decays of PGN.

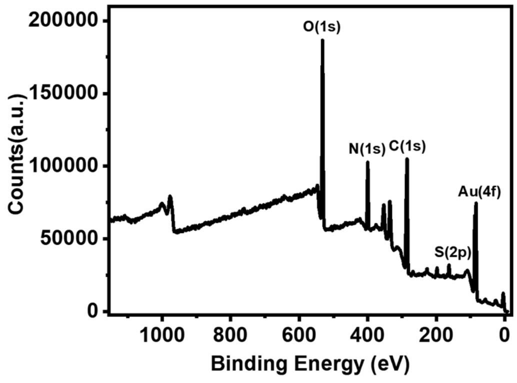

Figure S5. The whole XPS spectra of PGN.

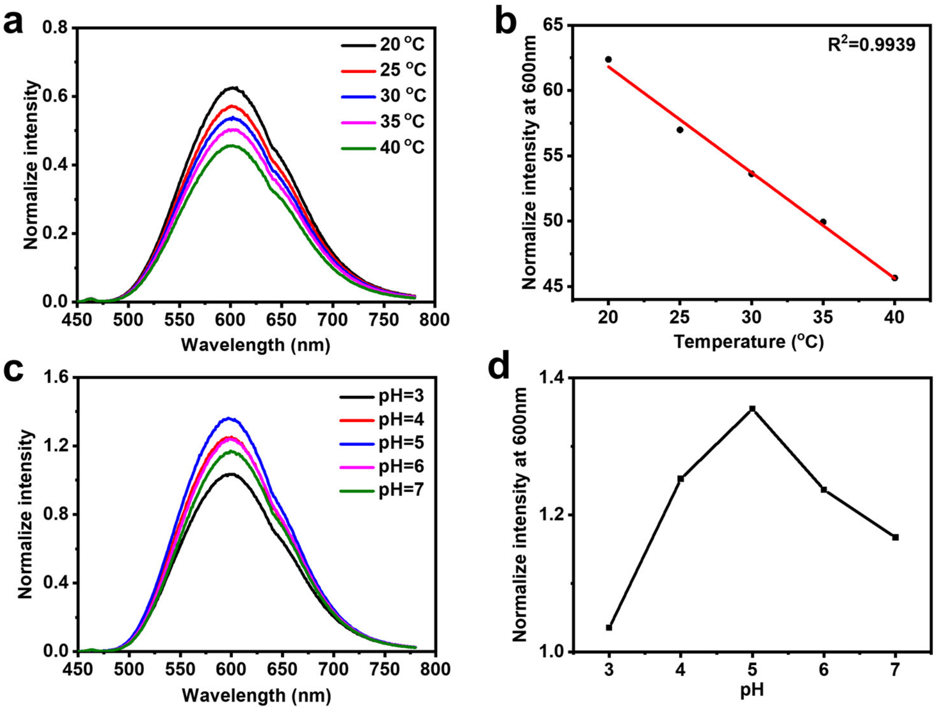

Figure S6. (a) Fluorescence spectra of PGN at different temperatures (from 20 to 40 °C). (b) Correlation of fluorescence intensity at 600 nm with temperature ranging from 20 to 40 °C. (c) Fluorescence spectra of PGN at different pH (from 3 to 7). (d) Correlation of fluorescence intensity at 600 nm with pH in the range of 3-7.

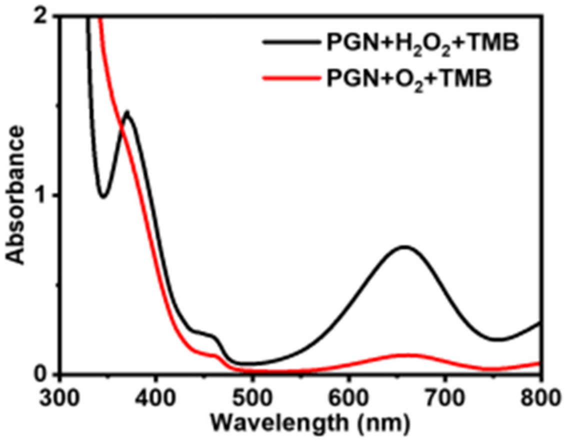

Figure S7. Comparison of the peroxidase-like (in presence of H_2O_2) and the oxidase-like (in presence of dioxygen) activity of PGN.

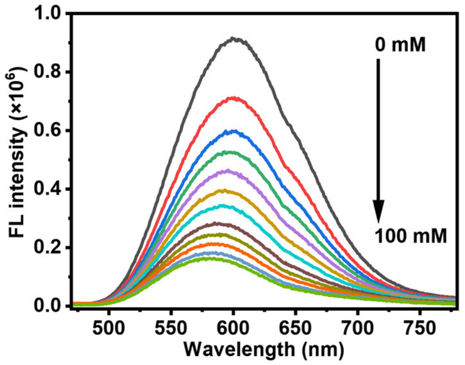

Figure S8. Fluorescence changes of the PGN-TMB system after adding H_2O_2 at different concentrations (0,5,10,20,30,40,50,60,70,80,90 and 100mM).

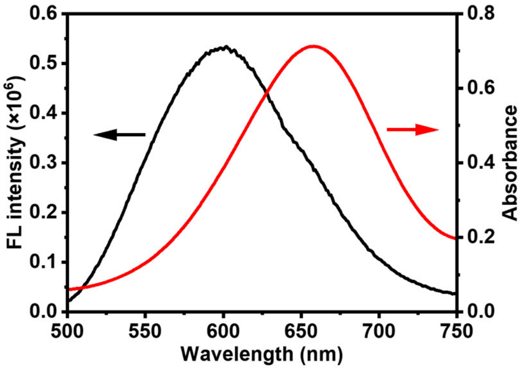

Figure S9. Emission spectra of PGN (black line) and UV-Vis absorption spectra of the PGN-TMB-H_2O_2 system (red line).

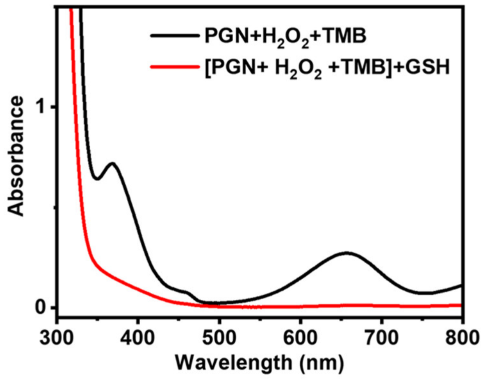

Figure S10. The UV-Vis spectrum of PGN+H_2O_2+TMB before and after adding GSH.

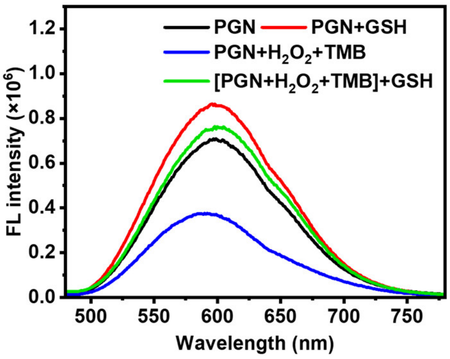

Figure S11. Fluorescence spectrum of PGN; PGN+GSH; PGN+H_2O_2+TMB and [PGN+H_2O_2+TMB] + GSH.

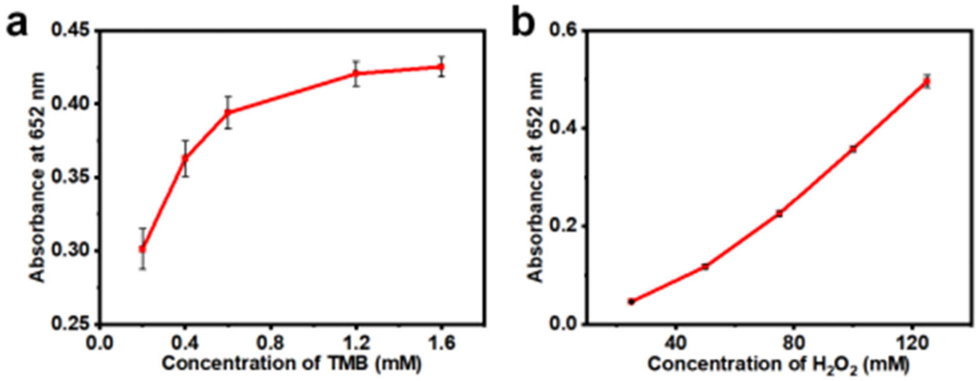

Figure S12. (a) The absorption peak at 652 nm in the presence of different concentrations of TMB (0.2, 0.4, 0.6, 1.2, 1.6 mM). (b) The absorption peak at 652 nm in the presence of different concentrations of H_2O_2 (25, 50, 75, 100, 125 mM). The concentration of the GSH-Au NCs was fixed at 100 μM (Au basis).

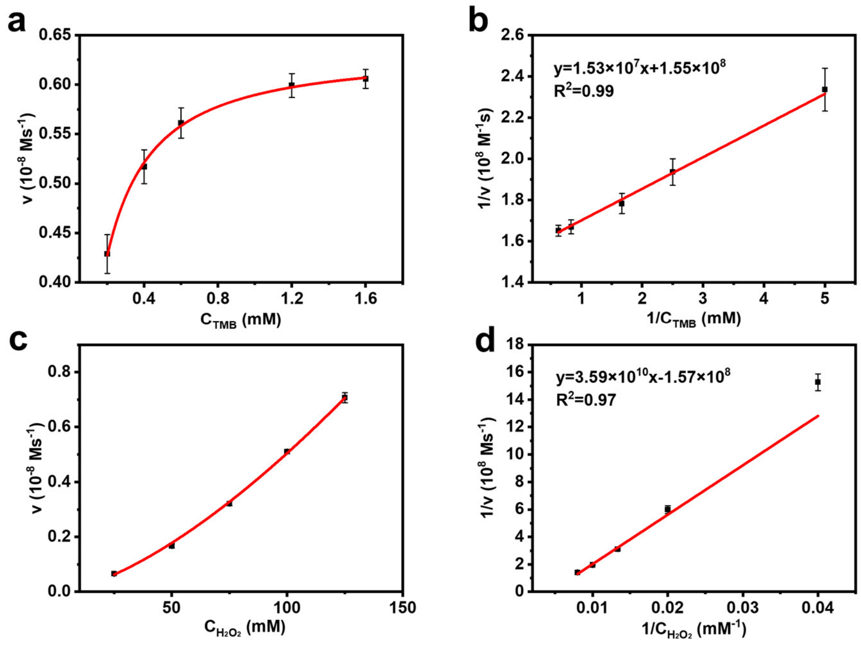

Figure S13. Steady-state kinetic assay and catalytic mechanism of the synthesized GSH-Au NCs towards various components: (a, b) 1 mM TMB and different concentrations of H_2O_2, (c, d) 100 mM H_2O_2 and different concentrations of TMB.

Table S1. Comparison of kinetic parameters of K_m and K_{cat} of PGN, and GSH-Au NCs.

Catalyst	Substrate	K_m (mM)	K_{cat} (s^{-1})	Reference
PGN	TMB	0.31	8×10^{-5}	This work
	H_2O_2	1069	5.35×10^{-4}	
GSH-Au NCs	TMB	0.0987	6.45×10^{-5}	[23]
	H_2O_2	228.66	6.37×10^{-5}	
